# Assessing the sensitivity of placental growth factor and soluble fms-like tyrosine kinase 1 at 36 weeks’ gestation to predict small-for-gestational-age infants or late-onset preeclampsia: a prospective nested case-control study

**DOI:** 10.1186/s12884-018-1992-x

**Published:** 2018-08-31

**Authors:** Teresa M. MacDonald, Chuong Tran, Tu’uhevaha J. Kaitu’u-Lino, Shaun P. Brennecke, Richard J. Hiscock, Lisa Hui, Kirsten M. Dane, Anna L. Middleton, Ping Cannon, Susan P. Walker, Stephen Tong

**Affiliations:** 1 Mercy Perinatal, Mercy Hospital for Women, Melbourne, VIC Australia; 2 Department of Obstetrics and Gynaecology, University of Melbourne, Melbourne, VIC Australia; 3 Translational Obstetrics Group, University of Melbourne, Melbourne, VIC Australia; 4 Department of Laboratory Services, Royal Children’s Hospital, Melbourne, VIC Australia; 5 Department of Maternal-Fetal Medicine, Royal Women’s Hospital, Melbourne, VIC Australia

**Keywords:** Biomarker, Fetal growth restriction, Late-onset, Placental growth factor, Preeclampsia, Small-for-gestational-age, Soluble fms-like tyrosine kinase 1

## Abstract

**Background:**

Fetal growth restriction is a disorder of placental dysfunction with three to four-fold increased risk of stillbirth. Fetal growth restriction has pathophysiological features in common with preeclampsia. We hypothesised that angiogenesis-related factors in maternal plasma, known to predict preeclampsia, may also detect fetal growth restriction at 36 weeks’ gestation. We therefore set out to determine the diagnostic performance of soluble fms-like tyrosine kinase 1 (sFlt-1), placental growth factor (PlGF), and the sFlt-1:PlGF ratio, measured at 36 weeks’ gestation, in identifying women who subsequently give birth to small-for-gestational-age (SGA; birthweight <10th centile) infants. We also aimed to validate the predictive performance of the analytes for late-onset preeclampsia in a large independent, prospective cohort.

**Methods:**

A nested 1:2 case-control study was performed including 102 cases of SGA infants and a matched group of 207 controls; and 39 cases of preeclampsia. We determined the diagnostic performance of each angiogenesis-related factor, and of their ratio, to detect SGA infants or preeclampsia, for a predetermined 10% false positive rate.

**Results:**

Median plasma levels of PlGF at 36 weeks’ gestation were significantly lower in women who subsequently had SGA newborns (178.5 pg/ml) compared to normal birthweight controls (326.7 pg/ml, *p* < 0.0001). sFlt-1 was also higher among SGA cases, but this was not significant after women with concurrent preeclampsia were excluded. The sensitivity of PlGF to predict SGA infants was 28.8% for a 10% false positive rate. The sFlt-1:PlGF ratio demonstrated better sensitivity for preeclampsia than either analyte alone, detecting 69.2% of cases for a 10% false positive rate.

**Conclusions:**

Plasma PlGF at 36 weeks’ gestation is significantly lower in women who subsequently deliver a SGA infant. While the sensitivity and specificity of PlGF currently limit clinical translation, our findings support a blood-based biomarker approach to detect late-onset fetal growth restriction. Thirty-six week sFlt-1:PlGF ratio predicts 69.2% of preeclampsia cases, and could be a useful screening test to triage antenatal surveillance.

**Electronic supplementary material:**

The online version of this article (10.1186/s12884-018-1992-x) contains supplementary material, which is available to authorized users.

## Background

Fetal growth restriction (FGR) due to placental insufficiency [1] is a major risk factor for stillbirth [2]. Small-for-gestational-age (SGA, birthweight <10th centile) fetuses, a common surrogate classification for FGR, have three to four-fold increased stillbirth risk at every gestation [2–4]. A large prospective cohort study demonstrated that when SGA fetuses are detected and appropriately managed, the rate of stillbirth is halved compared to pregnancies where a SGA fetus remains undiagnosed [2].

Improved identification of FGR has been listed as a top 10 priority to reduce the global burden of stillbirth [5]. Measuring symphysis-fundal height is current practice for detecting the SGA fetus, despite low sensitivity, reported at 17–58% over the last decade [6–8]. While universal third trimester ultrasound improves detection of SGA fetuses beyond that of selective ultrasound, its sensitivity is only 57%, with 35% positive predictive value [9]. A blood test able to detect the SGA fetus with better accuracy would provide clinicians with a valuable screening tool that may reduce the incidence of stillbirth.

Preeclampsia is associated with disordered release of angiogenesis-related factors into the maternal circulation – reduced pro-angiogenic placental growth factor (PlGF) and increased anti-angiogenic soluble fms-like tyrosine kinase 1 (sFlt-1) [10–12]. A high sFlt-1:PlGF ratio is associated with preeclampsia, performs as a better predictor than either analyte alone [13, 14] and demonstrates high negative predictive value [15, 16].

Like preeclampsia, FGR is characterised by placental dysfunction which can lead to aberrant release of angiogenesis-related factors into the maternal circulation. The sFlt-1:PlGF ratio has been shown to be significantly higher in cases of ultrasound-diagnosed SGA fetuses [17], and in women where SGA fetuses have failed to be detected by routine third trimester ultrasound [18]. A nested case-control study specifically including pregnant women with ultrasound estimated fetal weight > 10th centile at 32–36 weeks’ gestation, demonstrated significantly higher sFlt-1:PlGF ratios (at the same gestation as the ultrasound) among 80 cases of a term SGA infant compared to 80 controls, but with just 30% sensitivity at a 10% false positive rate (FPR) [18]. While a relationship between elevated sFlt-1:PlGF ratios and SGA fetuses has been described, the predictive value of the ratio at a single point in late pregnancy to identify the SGA, without ultrasound, has not been established.

Given that sFlt-1 and PIGF are placenta-derived proteins, we hypothesised that measuring their levels at 36 weeks’ gestation may be able to detect late-onset placental dysfunction to identify the SGA fetus, and to predict preeclampsia. We performed this nested case-control study from a large prospective cohort of 1000 pregnant women who had blood sampled at 36 weeks’ gestation. We examined the performance of sFlt-1, PlGF and the sFlt-1:PlGF ratio at 36 weeks’ gestation in the identification of women who subsequently gave birth to a SGA infant, or who developed preeclampsia.

## Methods

This analysis is part of the Fetal Longitudinal Assessment of Growth (FLAG) study at the Mercy Hospital for Women, a tertiary maternity hospital in Melbourne with approximately 6000 births annually. The FLAG study (https://mercyperinatal.com/project/fetal-studies), designed to identify biomarkers to detect SGA fetuses, included prospective collection of 2015 blood samples from pregnant women at 36 weeks’ gestation.

We performed a 1:2 nested case-control study using samples chosen from the first 1000 FLAG participants. We compared the 36 week sFlt-1, PlGF and sFlt-1:PlGF ratio values from women who delivered a SGA infant, to the analyte levels from a cohort of appropriate-for-gestational-age (AGA, birthweight ≥10th centile) controls, matched for maternal age, booking body mass index (BMI), smoking status, gestational diabetes mellitus (GDM), and parity. While a cohort study utilising all 1000 samples would be more powerful, and while case-control studies can be subject to overfitting, we used this nested case-control design to minimise costs. We made an a priori plan to proceed to a validation study utilising the remaining 1015 samples (from the subsequent FLAG study participants), which would mitigate the effects of any overfitting in the initial case-control study, if any analyte(s) demonstrated good diagnostic performance in predicting SGA infants.

This study was approved by the Mercy Health Research Ethics Committee (Ethics Approval Number R14/12) and written informed consent was obtained from all participants.

English-speaking women aged over 18 years, carrying a singleton pregnancy with normal mid-trimester morphology ultrasound were eligible to participate. Women booked to attend the Mercy Hospital for Women for their oral glucose tolerance test, offered around 28 weeks’ gestation to diagnose GDM, were screened for eligibility and invited to participate between January 2015 and September 2016. Women who consented formed a convenience series of participants. Samples from women where a SGA fetus or preeclampsia were suspected at the time of blood sampling were not excluded. Whole blood was collected in a 10 ml ethylenediaminetetraacetic acid tube at 35^+ 0^ to 37^+ 0^ weeks’ gestation inclusive. Plasma was stored at − 80 °C until the time of PlGF and sFlt-1 measurement.

### Outcomes and diagnostic criteria

Maternal characteristics and pregnancy outcomes were obtained from review of each participant’s medical record, investigation results and hospital database entry, by a single clinician blinded to sFlt-1 and PlGF levels. Similarly, scientists performing the measurement of sFlt-1 and PlGF levels were blinded to the clinical characteristics and birthweight centiles of the participants. All sFlt-1 and PlGF levels were measured for research purposes only, and were not made available to any clinician involved in participants’ obstetric care. Therefore there was no possibility of intervention bias.

Infant birthweights were assigned a customised centile using the GROW software [19] (http://www.gestation.net/), which generates a ‘term optimal weight’ based on an optimised fetal weight standard. We adjusted for the following non-pathological factors: maternal height, weight and parity; infant sex; and exact gestational age. Coefficients for the Australian dataset of GROW were informed by a local dataset; the multiple regression model has a constant to which weight is added or subtracted for each of the adjusted variables. SGA was defined as customised birthweight <10th centile.

Preeclampsia was diagnosed according to The American College of Obstetricians and Gynecologists’ Taskforce on Hypertension in Pregnancy definition [20]: new onset hypertension (blood pressure ≥ 140 mmHg systolic, or ≥ 90 mmHg diastolic on two occasions ≥ 4 h apart after 20 weeks’ gestation); plus one of new-onset: proteinuria, thrombocytopaenia, renal insufficiency, impaired liver function, pulmonary oedema or cerebral symptoms.

We analysed the differences in sFlt-1, PlGF and sFlt-1:PlGF ratio values between the control group and three different case groups: (i) ‘All SGA’ – all cases where the infant was SGA, including cases of concurrent preeclampsia; (ii) ‘SGA only’ – cases of concurrent preeclampsia were excluded; (iii) ‘Preeclampsia’ – all cases of preeclampsia, regardless of birthweight centile.

For cases of birthweight <10th centile, we also searched hospital ultrasound records to see which cases (i) were referred for a clinically-indicated third trimester ultrasound scan which included biometry to estimate fetal weight, and (ii) which cases were identified by a third trimester ultrasound as SGA on the basis of EFW or abdominal circumference < 10th centile. This allowed us to compare the sensitivities of the analytes to detect SGA to that of current clinical practice – selective ultrasound – in our institution.

### Assessment of plasma analyte levels

Maternal plasma levels of sFlt-1 and PlGF were measured with a commercial electrochemiluminescence immunoassay platform (Roche Diagnostics). Analysis of change in sFlt-1 and PlGF values over gestational age in days was made using LOWESS smooth and regression techniques of both mean and median values (Additional file 1: S2). There was no significant trend for either sFlt-1 or PlGF values seen across the gestational weeks where sampling was performed (35^+ 0^–37^+ 0^ weeks), hence adjustment for gestational age was not performed.

### Statistical analysis

Maternal characteristics and birth outcome data were compared for all women who delivered SGA infants, and for all cases of preeclampsia, against controls using unpaired t-test or rank-sum test for continuous data, according to distribution; and Chi-squared test for categorical data. Statistical analyses were performed using GraphPad Prism version 6 (GraphPad Software Inc., San Diego, CA) and Stata v14 (College Station, TX: StataCorp LP). Two-sided significance level was set at 0.05.

We determined the sensitivities, at a predetermined 10% FPR, of sFlt-1, PlGF, and the sFlt:PlGF ratio for the detection of: (i) SGA <10th centile, with and without concurrent preeclampsia, (ii) SGA <3rd centile, with and without concurrent preeclampsia, and (iii) Preeclampsia. Overall discrimination of the analytes for each disease group was assessed with area under the Receiver Operating Characteristic (ROC) curve analysis. In this nested case-control study positive predictive value (PPV) and negative predictive value (NPV) were calculated using the sampling fraction adjustment based upon the number of controls in this study compared to the original cohort and associated 95% confidence limits were calculated using a logit based standard error [21].

We chose 10% FPR as the cut-off as this is the FPR of universal ultrasound biometry at 36 weeks’ gestation, as determined by the large, prospective, blinded Pregnancy Outcome Prediction study [9]. We chose to assess for SGA <10th centile, as this is considered by many to be an important threshold clinically [22]. In addition, customised birthweight <10th centile was the threshold used to define FGR in the large population study of over 92,000 births that demonstrated a halving of stillbirth risk when fetuses below this threshold were detected antenatally [2]. We also evaluated the performance of the analytes to predict infants destined to be born at <3rd centile as this was a cut-off agreed upon by recent Delphi procedure as a consensus definition of FGR [23].

## Results

### Baseline characteristics

**Fig. 1:**
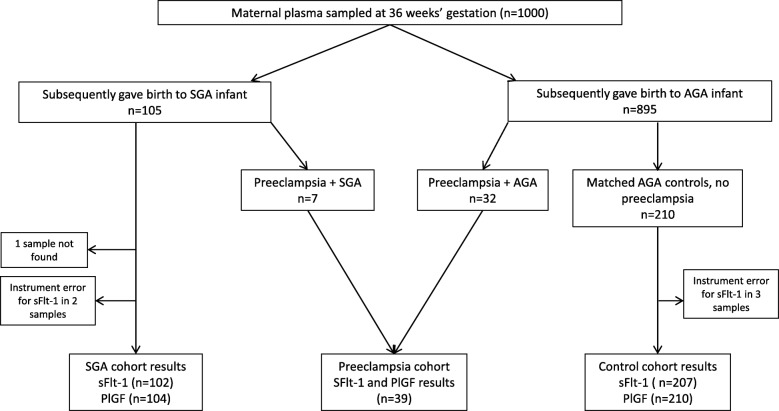
Study profile. AGA = appropriate-for-gestational-age (customised birthweight ≥10th centile), n = number, PlGF=Placental growth Factor, sFlt-1 = soluble fms-like tyrosine kinase 1, SGA = small-for-gestational-age (customised birthweight <10th centile)

Between March 2015 and February 2016 the first 1000 36 week FLAG study plasma samples were obtained. 105 (10.5%) participants delivered a SGA infant, including seven with co-existent preeclampsia. There were 28 (2.8%) cases of infant birthweight <3rd centile (one with concurrent preeclampsia). There were 32 cases of preeclampsia among women with AGA newborns (39 (3.9%) total preeclampsia cases). We matched the 105 women with SGA infants to 210 controls. Due to instrument error sFlt-1 values were not available for two SGA cases, and three controls (Fig. 1).

Maternal characteristics and birth outcomes for participants with complete sample analysis results are summarised in Table 1. There were no significant differences between controls and SGA cases except for birthweight, birthweight centiles and gestation at delivery: SGA infants were a mean 864 g and 46.8 centiles smaller, and were born 5 days’ gestation earlier than control infants. Participants who developed preeclampsia had significantly higher booking BMI than controls (mean 27.4 vs 25.4 kg/m^2^); and significantly higher emergency caesarean section rate. Infants of preeclamptic mothers were also significantly smaller than those of controls, and were born 5 days’ gestation earlier.

**Table 1:** Participant characteristics compared between controls and: (i) those with a SGA infant; (ii) women who developed preeclampsia

	Controls (*n* = 207)	All SGA (*n* = 102)	*P*	Preeclampsia (*n* = 39)	*P*
Age (years)	32.1 (4.7)	31.9 (4.2)	0.72	33.1 (4.7)	0.27
Booking Body Mass Index (kg/m^2^)	25.4 (4.6)	25.4 (5.8)	0.45	27.6 (6.0)	0.02
Smoking status					
Current smoker	17 (8.2%)	8 (7.8%)	0.98	2 (5.1%)	0.80
Ex-smoker	51 (24.6%)	26 (25.5%)		10 (25.6%)	
Never	139 (67.1%)	68 (66.7%)		27 (69.2%)	
Gestational diabetes mellitus	38 (18.4%)	18 (17.6%)	1.00	9 (23.1%)	0.51
Parity					
0	118 (57.0%)	58 (56.9%)	0.97	28 (71.8%)	0.20
1	65 (31.4%)	33 (32.4%)		9 (23.1%)	
> 1	24 (11.6%)	11 (10.8%)		2 (5.1%)	
Onset of delivery					
Induction of labour	88 (42.5%)	47 (46.1%)	0.08	19 (48.7%)	0.25
Spontaneous labour	96 (46.4%)	36 (35.3%)		13 (33.3%)	
No labour	23 (11.1%)	19 (18.6%)		7 (17.9%)	
Mode of delivery					
Normal vaginal delivery	102 (49.3%)	45 (44.1%)	0.64	11 (28.2%)	0.01
Instrumental delivery	42 (20.3%)	22 (21.6%)		7 (17.9%)	
Emergency caesarean	42 (20.3%)	20 (19.6%)		17 (43.6%)	
Elective caesarean	21 (10.1%)	15 (14.7%)		4 (10.3%)	
Birthweight (g)	3547 (444)	2683 (333)	< 0.0001	3308 (640)	0.005
Birthweight centile	52.1 (26.1)	5.3 (3.0)	< 0.0001	42.8 (30.4)	0.049
Gestational age at delivery (weeks^+days^)	39^+ 5^ (1^+ 1^)	39^+ 0^ (1^+ 4^)	< 0.0001	39^+ 0^ (1^+ 4^)	0.03

Data represented as mean (standard deviation), or number (%). *g* grams, *kg* kilograms, *m* metres, *n* number, *PlGF* placental growth factor, *sFlt-1* soluble fms-like tyrosine kinase 1, *SGA* small-for-gestational-age (birthweight <10th centile). Note: some percentages do not sum to 100% due to rounding to one decimal place

### Angiogenesis factor levels in cases compared to controls

Analysis was performed according to three case groups: (i) ‘All SGA’ – concurrent preeclampsia cases included; (ii) ‘SGA only’ – concurrent preeclampsia cases excluded; (iii) ‘Preeclampsia’ – all preeclampsia cases included, regardless of birthweight centile. Each case group was independently compared to controls (AGA infant, no preeclampsia).

(i) **‘All SGA’ vs controls**

**Fig. 2:**
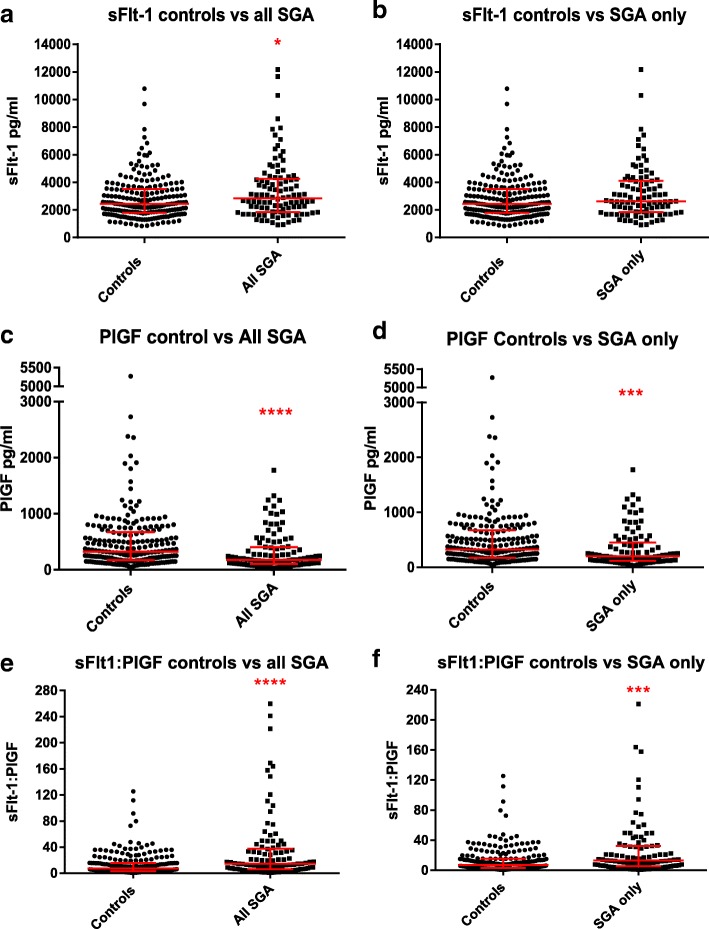
Levels of angiogenesis-related factors in cases of small-for-gestational-age (SGA) fetuses compared to controls. **a.** soluble fms-like tyrosine kinase-1 (sFlt-1) levels in ‘All SGA’ cases and controls; **b.** sFlt-1 levels in ‘SGA only’ (excluding concurrent preeclampsia) cases and controls; **c.** Placental growth factor (PlGF) levels in ‘All SGA’ cases and controls; **d.** PlGF levels in ‘SGA only’ cases and controls; **e.** sFlt-1:PlGF ratios in ‘All SGA’ cases and controls; **f.** sFlt-1:PlGF ratios in ‘SGA only’ cases and controls. Medians and interquartile ranges shown. * = *P* < 0.05, *** = *P* < 0.001, **** = *P* < 0.0001

The angiogenesis factors were all significantly altered in cases of SGA infants compared to controls (Fig. 2a, c, e, Table 2). When comparing ‘All SGA’ to controls, the median sFlt-1 level was significantly higher and the median PlGF level was significantly lower. Correspondingly, the median sFlt-1:PlGF ratio of the ‘All SGA’ cohort was significantly higher than that of controls (14.24 vs 7.11 respectively, *P* < 0.0001).

(ii) **‘SGA only’ vs controls**

**Table 2:** sFlt-1, PlGF and sFlt-1:PlGF ratios compared between controls and: (i) cases with a SGA infant; (ii) preeclampsia cases

	Controls	All SGA	*P*	SGA only	*P*	Preeclampsia	*P*
sFlt-1 (pg/ml)	2446 [1795–3503]	2837 [1850–4250]	0.02	2620 [1835–4099]	0.10	4857 [3777–6941]	< 0.001
PlGF (pg/ml)	326.7 [173.1–675.4]	178.5 [106.4–404.8]	< 0.001	199.9 [118.1–448.6]	< 0.001	99.73 [61.99–158.6]	< 0.001
sFlt-1:PlGF ratio	7.11 [3.08–15.58]	14.24 [6.00–37.37]	< 0.001	13.00 [5.27–32.44]	< 0.001	54.25 [26.81–114.5]	< 0.001

Data presented as median [25th–75th percentile]. “All SGA” = Cases with a SGA fetuses including cases with co-existent preeclampsia; “SGA only” = Cases with SGA fetuses with cases of co-existant preeclampisa excluded; pg/ml = picogram/millilitre, *PlGF* placental growth factor, *sFlt-1* soluble fms-like tyrosine kinase-1, *SGA* small-for-gestational-age (birthweight <10th centile). For sFlt-1 and sFlt-1:PlGF ratio results n = 207 for controls, n = 102 for ‘All SGA’, n = 39 for ‘Preeclampsia’. For PlGF results *n* = 210 for controls, *n* = 104 for ‘All SGA’, and *n* = 39 for ‘Preeclampsia’

When the angiogenesis factor levels were compared between controls and cases of ‘SGA only’ (excluding cases of co-existent preeclampsia) the differences between the groups became less pronounced (Fig. 2, Table 2). The median PlGF level remained significantly lower in ‘SGA only’ cases compared to controls, but there was no significant difference in sFlt-1 levels. The median sFlt-1:PlGF ratio of the ‘SGA only’ cases remained significantly higher than that of the controls (13.00 vs 7.11 respectively, *p* = 0.0006).

(iii) **Preeclampsia versus controls**

**Fig. 3:**
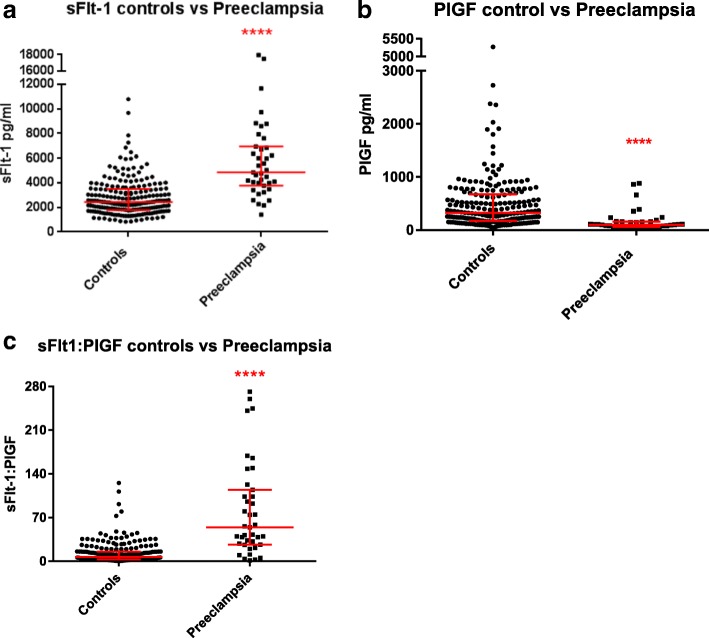
Levels of angiogenesis-related factors in cases of preeclampsia compared to controls. **a.** soluble fms-like tyrosine kinase-1 (sFlt-1) levels in preeclampsia cases and controls; **b.** Placental growth factor (PlGF) levels in preeclampsia cases and controls; **c.** sFlt-1:PlGF ratios in preeclampsia cases and controls. Medians and interquartile ranges shown. **** = *P* < 0.0001

The levels of the angiogenesis factors were all significantly different in cases of preeclampsia compared to controls (Fig. 3, Table 2). When comparing preeclampsia cases to controls, the median sFlt-1 level was significantly higher, and median PlGF was significantly lower. The median sFlt-1:PlGF ratio of the preeclampsia cohort was correspondingly significantly higher than that of the control group (54.25 vs 7.11 respectively, *P* < 0.0001).

### Sensitivity of the analytes to detect the SGA and preeclampsia

**Fig. 4:**
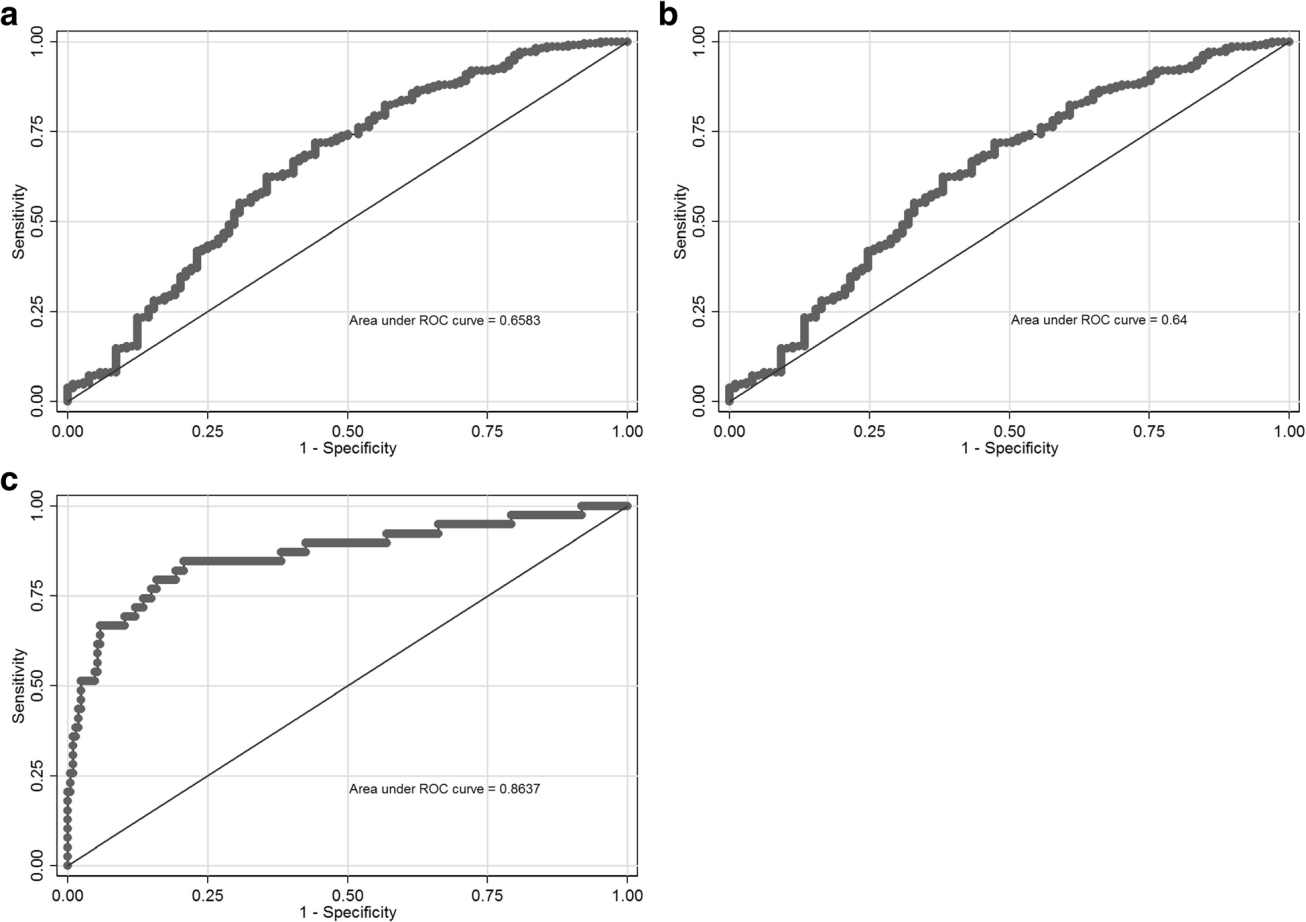
Receiver operating characteristic curves for most sensitive analytes to detect small-for-gestational-age (SGA) infants, or preeclampsia. **a.** ROC curve for the performance of placental growth factor (PlGF) in detecting ‘All SGA’ – including cases of concurrent preeclampsia; **b.** ROC curve for the performance of PlGF in detecting ‘SGA only’ – excluding cases of concurrent preeclampsia; **c.** ROC curve for the performance of the soluble fms-like tyrosine kinase 1:PlGF ratio in detecting preeclampsia

The sensitivities of the analytes at a fixed 10% FPR (90% specificity) to predict birth of a SGA infant, or preeclampsia, are presented in Table 3. In Fig. 4 we have also presented the area under the ROC curve as a measure of overall discrimination for the analyte that had the greatest sensitivity for each of ‘All SGA’, ‘SGA only’ and ‘Preeclampsia’.

**Table 3:** Diagnostic performance of each analyte to predict <10th and < 3rd centile infants, and preeclampsia

Outcome	Analyte and cut-point	Sensitivity	Specificity	PPV	NPV
Birthweight < 10th centile (All SGA)	sFlt-1 > 4570 pg/ml	19.6% [12.4–28.6%]	89.9% [84.9–93.6%]	18.0% [11.1–27.9%]	90.8% [89.8–91.6%]
	PlGF < 117.3 pg/ml	28.8% [20.4–38.6%]	89.5% [84.6–93.3%]	24.2% [16.3–34.5%]	91.6% [90.5–92.5%]
	sFlt-1:PlGF > 33.4	26.5% [18.2–36.1%]	89.9% [84.9–93.6%]	22.9% [15.0–33.2%]	91.5% [90.5–92.4%]
Birthweight < 10th centile (SGA only)	sFlt-1 > 4570 pg/ml	16.8% [9.9–25.9%]	89.9% [84.9–93.6%]	14.8% [8.7–24.2%]	91.1% [90.3–91.9%]
	PlGF < 117.3 pg/ml	24.7% [16.5–34.5%]	89.5% [84.6–93.3%]	20.2% [13.0–30.0%]	91.7% [90.7–92.6%]
	sFlt-1:PlGF > 33.4	22.1% [14.2–31.8%]	89.9% [84.9–93.6%]	18.6% [11.6–28.5%]	91.7% [90.7–92.5%]
Birthweight < 3rd centile (All SGA)	sFlt-1 > 4570 pg/ml	21.4% [8.3–41.0%]	87.5% [83.1–91.2%]	4.7% [2.2–9.7%]	97.5% [96.9–97.9%]
	PlGF < 117.3 pg/ml	32.1% [15.9–52.4%]	85.0% [80.3–88.9%]	5.8% [3.3–10.1%]	97.8% [97.1–98.3%]
	sFlt-1:PlGF > 33.4	28.6% [13.2–48.7%]	85.8% [81.1–89.6%]	5.5% [2.9–10.0%]	97.7% [97.0–98.1%]
Birthweight < 3rd centile (SGA only)	sFlt-1 > 4570 pg/ml	22.2% [8.6–42.3%]	88.7% [84.4–92.2%]	5.2% [2.4–10.7%]	97.6% [97.1–98.1%]
	PlGF < 117.3 pg/ml	29.6% [13.8–50.2%]	86.4% [81.9–90.2%]	5.7% [3.1–10.4%]	97.8% [97.2–98.3%]
	sFlt-1:PlGF > 33.4	25.9% [11.1–46.3%]	87.3% [82.7–91.0%]	5.4% [2.7–10.3%]	97.7% [97.1–98.2%]
Preeclampsia	sFlt-1 > 4570 pg/ml	56.4% [39.6–72.2%]	89.9% [84.9–93.6%]	18.4% [12.1–26.9%]	98.1% [97.3–98.6%]
	PlGF < 117.3 pg/ml	64.1% [47.2–78.8%]	89.5% [84.6–93.3%]	19.9% [13.6–28.2%]	98.4% [97.6–98.9%]
	sFlt-1:PlGF > 33.4	69.2% [52.4–83.0%]	89.9% [84.9–93.6%]	21.7% [14.9–30.4%]	98.6% [97.8–99.1%]

Data presented with [95% Confidence Interval]. *determined by a false positive rate of 10% in the birthweight ≥10th centile and no preeclampsia control group. “All SGA” = Cases of SGA fetuses including cases with co-existent preeclampsia; “SGA only” = Cases of SGA fetuses with cases of co-existant preeclampisa excluded; *NPV* Negative Predictive value, pg/ml = pictogram/millilitre, *PlGF* placental growth factor, *PPV* Positive Predictive Value, *sFlt-1* soluble fms-like tyrosine kinase-1, *SGA* small-for-gestational-age

For ‘All SGA’ and ‘SGA only’, at both <10th and < 3rd centile definitions, sFlt-1 had sensitivities below 23%. PlGF was the most sensitive analyte to detect both ‘All SGA’ and ‘SGA only’, but with sensitivities less than 33%. For ‘All SGA’ and ‘SGA only’ at both <10th and < 3rd centile definitions, the sFlt-1:PlGF ratio demonstrated less than 29% sensitivity (Table 3). For PIGF, the area under the ROC curve was only 0.66 for ‘All SGA’; with a similarly modest result for ‘SGA only’ (Fig. 4a & b). Given these very modest performances for the detection of SGA fetuses, we did not think it would be cost-effective or valuable to proceed with the validation step in our second 1015 samples, so we did not perform it.

Seventy four (70.4%) of the 105 cases of a SGA infant were referred for a third trimester ultrasound to estimate fetal weight (41 were performed between 35^+ 0^ and 37^+ 0^ weeks – the same gestation range as our blood samples). 24 (32.4%) of the 74 third trimester ultrasound scans detected a SGA fetus with an EFW or abdominal circumference measurement <10th centile. Therefore, the strategy of selective ultrasound, in practice in our institution, performed with a sensitivity for SGA of 22.9% (24 out of 105 cases identified) overall.

All three analytes demonstrated moderate sensitivity, at over 56% for a 10% FPR, for preeclampsia (Table 3). The sFlt-1:PlGF ratio had the highest sensitivity for preeclampsia, at 69.2% with a 10% FPR. The sFlt-1:PlGF ratio performed moderately well on ROC curve analysis, with an area under the curve of 0.86 (Fig, 4c).

## Discussion

The central role of the placenta in preeclampsia and FGR suggests that these two clinical conditions may share common plasma biomarkers. We therefore investigated the value of two known placenta-derived preeclampsia biomarkers (and their ratio), for the prediction of SGA infants in a large, independent, prospective cohort. Through analysis of samples obtained from a large unselected cohort, we found maternal plasma PlGF at 36 weeks’ gestation to be significantly lower in women that subsequently gave birth to SGA infants compared to women with AGA newborns.

At a fixed 10% FPR, low PlGF identified 32.1% of women who subsequently gave birth to a newborn with birthweight <3rd centile, and 28.8% of those with a < 10th centile infant. Current tools for detection of late-onset FGR are poor. Significantly, a single PlGF level, even at the modest detection rates reported here, may still outperform the traditional methods of symphysis-fundal height [6], and selective ultrasound [9]. Indeed in our cohort, a strategy of selective ultrasound, the current clinical practice in our institution, predicted only 22.9% of SGA infants – comparatively less than PlGF would have. While the predictive performance of PlGF alone is not sufficient to warrant adoption into clinical practice, our data supports the potential of a multi-biomarker approach. We also report that sFlt-1 is not significantly altered in pregnancies with a SGA fetus unless preeclampsia is present, suggesting sFlt-1 to be a biomarker more specific to preeclampsia.

Our study also validated the diagnostic performance of the sFlt-1:PlGF ratio at 36 weeks’ gestation for late-onset preeclampsia. When we applied the same ratio cut-off (≥38 pg/ml) as used in the PROGNOSIS study [15] to our cohort, 66.7% sensitivity and 94.2% specificity were achieved (Table 4), surpassing the performance reported in the original trial for preeclampsia within 4 weeks of testing [15]. Application of the ≥38 pg/ml cut-off did not perform as well to predict SGA infants however, displaying sensitivities of 24.5% and 25.0% for <10th and < 3rd centile birthweight respectively (Table 4). Overall, the data from our study acts to validate some of the results from the PROGNOSIS study in an independent cohort, strengthening the potential clinical utility of the ratio for detecting late-onset preeclampsia.

**Table 4:** Diagnostic performance of sFlt-1:PlGF ratio ≥ 38 for preeclampsia, and < 10th and < 3rd centile infants

Outcome	Sensitivity	Specificity	PPV	NPV
Preeclampsia	66.7% [49.8–80.9%]	94.2% [90.1–97.0%]	31.8% [20.5–45.8%]	98.6% [97.8–99.1%]
Birthweight < 10th centile (All SGA)	24.5% [16.5–34.0%]	94.2% [90.1–97.0%]	32.4% [20.1–47.8%]	91.7% [90.7–92.5%]
Birthweight < 3rd centile (All SGA)	25.0% [10.7–44.9%]	89.3% [85.1–92.7%]	6.3% [3.2–12.3%]	97.6% [97.1–98.1%]

Data presented with [95% Confidence Interval]. *NPV* Negative Predictive value, *PlGF* placental growth factor, *PPV* Positive Predictive Value, *sFlt-1* soluble fms-like tyrosine kinase-1, *SGA* small-for-gestational-age

The strength of this study was our large prospective cohort of 1000 singleton pregnancies from which case-control samples were selected. Previous studies have investigated the relationships between sFlt-1 and/or PlGF and late-onset FGR, but most have utilized methods not readily translated into clinical practice for a general antenatal population. These methods include placental rather than plasma protein analysis [24]; multi-modality integrated models [25, 26]; prior ultrasound diagnosis of SGA [27–33]; and investigation confined to cases of preterm infants [32]. Our large cohort specifically enabled us to compare the utility of sFlt-1, PlGF, and their ratio, in all cases of a SGA infant as well as in a ‘SGA only’ cohort, actively removing the impact of participants with concurrent preeclampsia. Our results add clarity where previous findings have been inconsistent. Prior to this study, elevated sFlt-1 had been associated with FGR in some animal [34], and human [31, 32, 35, 36] studies with cases of preeclampsia excluded, but not in others [18, 33]. The large numbers in our study and the ability to test levels in both ‘All SGA’ and ‘SGA only’ cohorts have allowed us to confidently define sFlt-1 as a biomarker more specific to preeclampsia, rather than FGR.

Previous studies have measured the analytes at a variety of gestations, including first [26, 37] and second trimesters [38], in a longitudinal fashion [12], and at delivery [31, 39]. However, early measurement of the angiogenic factors is not predictive of late-onset disease [38], except if included in a complex model incorporating several maternal, ultrasonographic and blood-based risk factor assessments [26] – difficult to incorporate into clinical practice. In preeclampsia, the predictive performance of the analytes is improved when measured closer to the onset of disease [15]. Future biomarkers requiring only single blood sampling at 36 weeks’ gestation would have the potential to be rapidly incorporated into clinical practice, with the availability of safe, acceptable interventions (surveillance and planned timely delivery) to reduce stillbirth risk for SGA fetuses.

While the predictive potential of a single measurement of the sFlt-1:PlGF ratio at 36 weeks’ gestation has been assessed for preeclampsia [16], only the Pregnancy Outcome Prediction (POP) study has previously analysed the sFlt-1:PlGF ratio specifically at 36 weeks’ gestation for FGR [40]. This study defined FGR as birthweight <10th centile plus perinatal morbidity and/or preeclampsia and found the combination of sFlt-1:PlGF ratio > 38 and ultrasound EFW <10th centile – both present together in just 3% of participants – to demonstrate a higher PPV (21.6%) and specificity (98%) than either parameter alone, but with lower sensitivity (only 38%) compared to either test alone. In this study, a 36 week PlGF value among the lowest decile performed with almost exactly the same diagnostic accuracy as the highest decile of the sFlt-1:PlGF ratio to identify FGR. Both tests performed with 43.1% sensitivity, 90.6% specificity, 6.7% PPV and 99% NPV for the definition of FGR in use [40]. This mirrors the finding in our study, that the addition of sFlt-1 to PlGF in the form of the ratio at 36 weeks’ gestation adds no value. It is also not surprising that the sensitivities reported for the analytes alone were higher in the POP study compared to ours – as the POP definition of FGR occurred in only 1.5% of the population. Interestingly, in the POP cohort, sFlt-1:PlGF ratio > 38 demonstrated 37.2% sensitivity for birth weight < 3rd centile. This was much higher than the 25% sensitivity seen in our study. However, the POP study utilised a large cohort study in comparison to our nested case-control design; and used a population reference to define birthweight centile in contrast to customised birth weight centiles.

One other previous study measured sFlt-1 and PlGF at 35–37 weeks’ gestation and reported their ability, in combination with ultrasound biometry and maternal characteristics, to predict SGA infants in the absence of preeclampsia, but did not assess the sFlt-1:PlGF ratio [36]. Again, similar to our findings, while addition of PlGF to biometry and maternal characteristics marginally improved the detection of SGA fetuses, addition of sFlt-1 did not [36]. Measurement of the analytes at a single time-point in late pregnancy was a strength of our study, adding to this body of evidence, as was our direct comparison of the sFlt-1:PlGF ratio to its constituent analytes.

The generalisability of our data is supported by the incidence of both preeclampsia and SGA infants among our participants, in keeping with expected rates [9, 41–43]. We present a well-characterised cohort, featuring a control group carefully matched for factors known to influence fetal growth. We used GROW software [19] to customise birthweight centiles, as this customised standard has stronger associations with adverse perinatal outcomes attributable to placental insufficiency than population references [44]. Additionally, the Elecsys immunoassays used for sFlt-1 and PlGF (Roche Diagnostics) have received Conformité Européene marking for use as in vitro medical devices.

One potential reason that PlGF displayed relatively low detection of SGA infants is that ‘SGA’ is not a functional diagnosis, and therefore may include infants who are constitutionally, rather than pathologically, small. Constitutionally small fetuses would not be expected to exhibit a low PlGF, as they are not suffering placental insufficiency. This in part may explain why PlGF levels, although significantly different, were not able to differentiate between cases and controls with both high sensitivity and specificity. However, PlGF was also not able to predict <3rd centile infants, those largely regarded to be growth restricted [23], with great accuracy which suggests that while low PlGF is associated with placental insufficiency, the strength of association is not strong enough for its use as a biomarker in isolation.

Overall, our study demonstrates that PlGF may be a useful component of a multi-biomarker predictive blood test for SGA, which we aim to develop. Our sFlt-1 results highlight that FGR and preeclampsia do not share the same plasma biomarker profile, such that future studies dedicated to FGR-specific biomarkers are needed. However, there are many challenges to performing well-conducted prospective studies, including the large numbers of participants required, and substantial associated costs. Despite this, we believe that future biomarkers requiring only single blood sampling at 36 weeks’ gestation would have the potential to be rapidly incorporated into clinical practice, with the availability of safe, acceptable interventions (surveillance and planned timely delivery) to reduce stillbirth risk for SGA fetuses.

## Conclusions

Thirty-six week plasma PlGF is significantly lower in women who subsequently deliver a SGA infant, but its diagnostic performance limits clinical translation as an individual biomarker. Further research is required to identify other biomarkers for FGR that together may perform with high sensitivity and specificity. The sFlt-1:PlGF ratio at 36 weeks however predicts preeclampsia with 69.2% sensitivity for 90% specificity, and therefore may be useful in triaging antenatal surveillance.

## Additional files

Additional file 1: **S1.** Assessment for variation in analytes due to gestation. (DOCX 12070 kb)

Additional file 2: **S2.** Study data. (XLSX 104 kb)

## Abbreviations

AGA: Appropriate-for-gestational-age (birthweight ≥10th centile)
BMI: Body Mass Index
FGR: Fetal growth restriction
FLAG: Fetal Longitudinal Assessment of Growth
FPR: False positive rate
g: Grams
GDM: Gestational Diabetes Mellitus
kg: Kilograms
m: Metres
n: Number
NPV: Negative Predictive Value
PlGF: Placental growth factor
PPV: Positive Predictive Value
ROC: Receiver Operating Characteristic
sFlt-1: Soluble fms-like tyrosine kinase 1
SGA: Small-for-gestational-age (birthweight <10th centile)
