# Supplementary material for: Assessing the sensitivity of placental growth factor and soluble fms-like tyrosine kinase 1 at 36 weeks’ gestation to predict small-for-gestational-age infants or late-onset preeclampsia: a prospective nested case-control study
Source: BMC Pregnancy Childbirth. 2018 Aug 31;18:354. doi: 10.1186/s12884-018-1992-x (PMC6119271; doi:10.1186/s12884-018-1992-x)
Supplement: Supplementary file 1 — S1. Assessment for variation in analytes due to gestation. (DOCX 12070 kb) [file 12884_2018_1992_MOESM1_ESM.docx]

**Assessment for variation in analytes due to gestation**

Assessing control group change

Dataset has 207 control patients. Analysis of change over gestational time measured in days was made for sflt1 & PLGF using loess smooth and regression both mean & median.

SFLT1


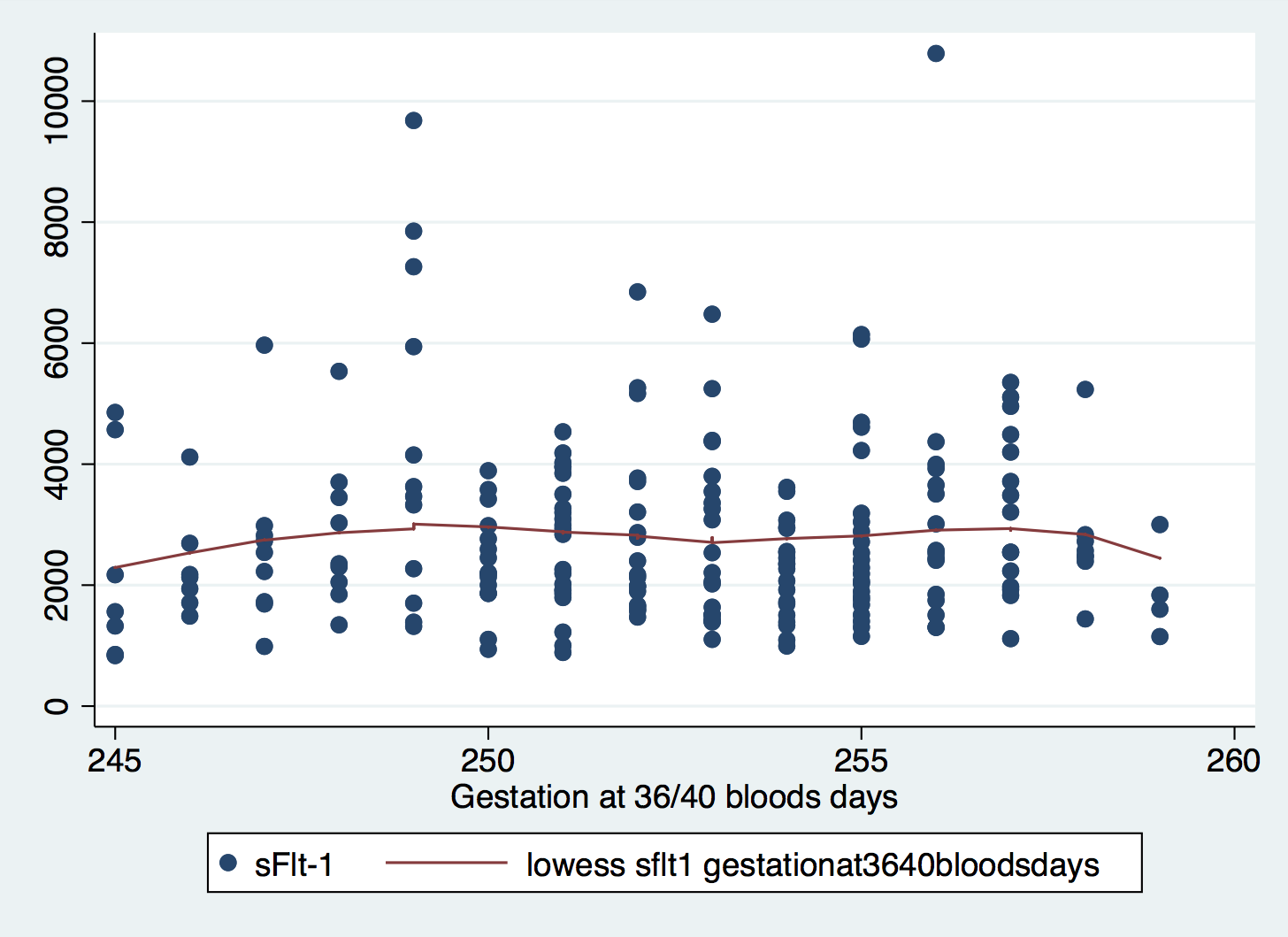


Source | SS df MS Number of obs = 207

-------------+---------------------------------- F(1, 205) = 0.03

Model | 69337.3732 1 69337.3732 Prob > F = 0.8621

Residual | 469725893 205 2291345.82 R-squared = 0.0001

-------------+---------------------------------- Adj R-squared = -0.0047

Total | 469795231 206 2280559.37 Root MSE = 1513.7

-------------------------------------------------------------------------------------------

sflt1 | Coef. Std. Err. t P>|t| [95% Conf. Interval]

--------------------------+----------------------------------------------------------------

gestationat3640bloodsdays | 5.220944 30.01309 0.17 0.862 -53.95297 64.39485

_cons | 1488.832 7576.063 0.20 0.844 -13448.16 16425.82

-------------------------------------------------------------------------------------------

Median regression Number of obs = 207

Raw sum of deviations 109869.6 (about 2446)

Min sum of deviations 109381 Pseudo R2 = 0.0044

-------------------------------------------------------------------------------------------

sflt1 | Coef. Std. Err. t P>|t| [95% Conf. Interval]

--------------------------+----------------------------------------------------------------

gestationat3640bloodsdays | 30.61538 30.73346 1.00 0.320 -29.97881 91.20958

_cons | -5328.769 7757.903 -0.69 0.493 -20624.28 9966.739

-------------------------------------------------------------------------------------------

There is no evidence for a trend in SFLT1 values over the sampling gestation found in this dataset.

PLGF


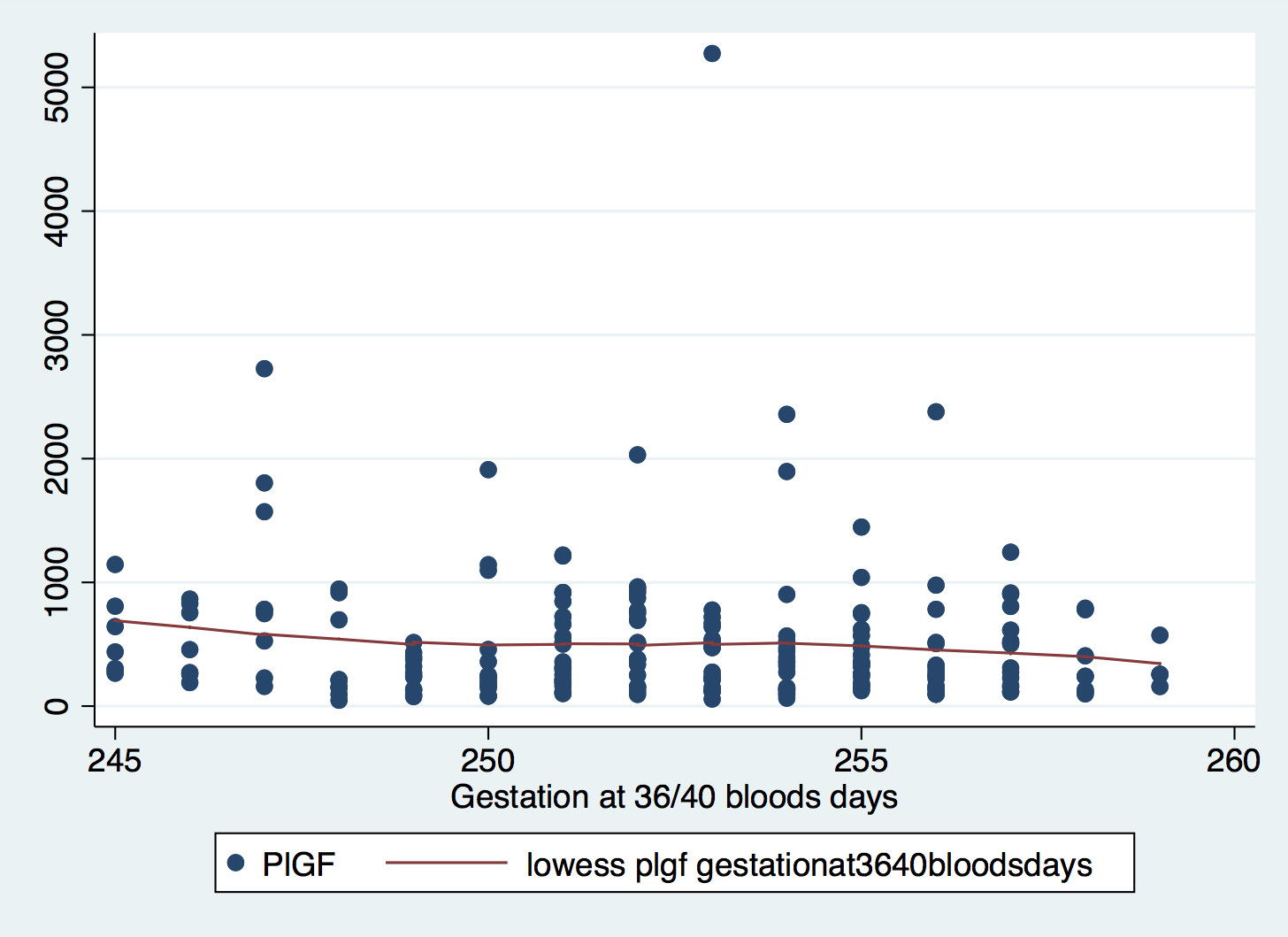


Source | SS df MS Number of obs = 207

-------------+---------------------------------- F(1, 205) = 1.23

Model | 373644.766 1 373644.766 Prob > F = 0.2693

Residual | 62441426.5 205 304592.324 R-squared = 0.0059

-------------+---------------------------------- Adj R-squared = 0.0011

Total | 62815071.2 206 304927.53 Root MSE = 551.9

-------------------------------------------------------------------------------------------

plgf | Coef. Std. Err. t P>|t| [95% Conf. Interval]

--------------------------+----------------------------------------------------------------

gestationat3640bloodsdays | -12.11978 10.94271 -1.11 0.269 -33.69447 9.454901

_cons | 3559.785 2762.217 1.29 0.199 -1886.211 9005.781

-------------------------------------------------------------------------------------------

Median regression Number of obs = 207

Raw sum of deviations 32523.99 (about 325.2)

Min sum of deviations 32420.99 Pseudo R2 = 0.0032

-------------------------------------------------------------------------------------------

plgf | Coef. Std. Err. t P>|t| [95% Conf. Interval]

--------------------------+----------------------------------------------------------------

gestationat3640bloodsdays | -9.8 8.676973 -1.13 0.260 -26.90755 7.307551

_cons | 2817 2190.288 1.29 0.200 -1501.379 7135.379

-------------------------------------------------------------------------------------------

There is no evidence for a trend in PLGF values over the sampling gestation found in this dataset.
